# Comparative Study of RP-HPLC and UV Spectrophotometric Techniques for the Simultaneous Determination of Amoxicillin and Cloxacillin in Capsules

**DOI:** 10.4103/0975-1483.63168

**Published:** 2010

**Authors:** Do T Giang, Vu D Hoang

**Affiliations:** 1 *Department of Analytical Chemistry and Toxicology, Hanoi University of Pharmacy 13-15 Le Thanh Tong, Hanoi, Vietnam*

**Keywords:** Absorbance ratio, amoxicillin, capsules, cloxacillin, compensation, HPLC, UV derivative spectrophotometry

## Abstract

Reversed-phase HPLC and UV spectrophotometric techniques using water as solvent have been developed and validated for the simultaneous determination of amoxicillin and cloxacillin in capsules. For both techniques, the linearity range of 60.073x2013;140.0 *µ*g/mL was studied. The spectrophotometric data show that non-derivative techniques, such as absorbance ratio and compensation, and ratio spectra first-order derivative could be successfully used for the co-assay of amoxicillin and cloxacillin. Based on the statistical comparison of spectrophotometric and chromatographic data, the interchangeability between HPLC and UV spectrophotometric techniques has been suggested for the routine analysis.

## INTRODUCTION

**Figure 1:**
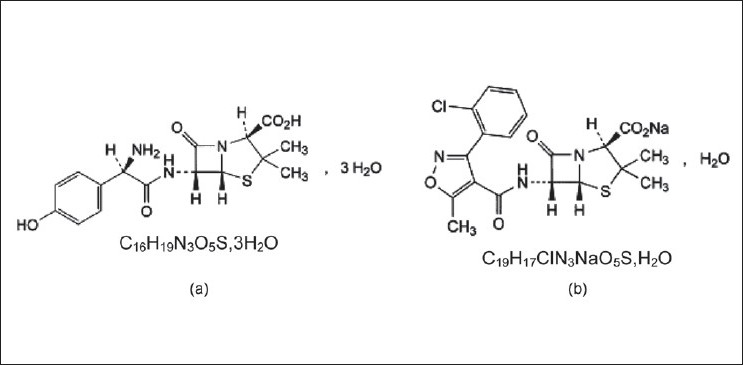
Chemical structures of (a) Amoxicillin trihydrate and (b) Cloxacillin sodium

Amoxicillin, formerly amoxycillin [Figure 1 a], is a moderate-spectrum beta-lactam antibiotic used to treat infections caused by penicillin-sensitive gram-positive bacteria as well as some gram-negative bacteria.[1] Amoxicillin is resistant to inactivation by gastric acid. It is usually the drug of choice because it is more rapidly and more completely absorbed than other beta-lactam antibiotics when orally administered. To overcome its sensitivity to destruction by beta-lactamases, amoxicillin has been co-administered with clavulanic acid, a potent betalactamase inhibitor[2] in pharmaceutical preparations.

Cloxacillin is a semisynthetic penicillin used as the sodium salt [Figure 1 b] to treat staphylococcal infections due to penicillinase-positive organisms.[1] Unlike amoxicillin, this antibiotic is incompletely absorbed from the gastrointestinal tract, and absorption is reduced by the presence of food in the stomach. To produce a wider spectrum of activity, cloxacillin may be co-formulated with other antibacterials, in particular with amoxicillin (ratio 1:1, w/w) in capsules.

In the literature, the high performance liquid chromatography (HPLC) technique has been reviewed as a valuable tool for the analysis of antibiotics in formulated and unformulated samples.[3] As a result, this technique has been widely used for the simultaneous determination of penicillins such as amoxicillin and cloxacillin in pharmaceuticals, biological fluids, and tissues.[4–8]

On the other hand, amoxicillin was also spectrophotometrically analyzed without prior separation using UV derivative techniques in the combination with clavulanic acid[9–12] or in antibiotic pharmaceutical mixtures.[13–16]

Albeit HPLC is often an official method for the analysis of antibiotics, the need for other simple, reproducible and accurate analytical methods still exists. Thus, this study was carried out to compare HPLC and UV spectrophotometric techniques using water only as solvent for the simultaneous determination of amoxicillin and cloxacillin in their combined capsules.

## EXPERIMENTAL DETAILS

### Apparatuses and conditions

A UNICAM UV 300 double beam spectrophotometer (Thermo Spectronic, USA) with a fixed slit width (1.5 nm) connected to an IBM computer loaded with Thermo Spectronic VISION32 software and 1 cm quartz cell were used for the registration and treatment of absorption spectra. For all solutions, zero-order spectra were recorded over the range from 200.0 to 300.0 nm against a blank (water) at Intelliscan mode to enhance the signal-to-noise ratio of absorbance peaks without extended scan duration with a ∆λ = 0.1 nm (i.e. 30-120 nm/min). To get the best signal-to-noise ratio and resolution, spectra and their corresponding derivative ones were further smoothed by using Savitzky – Golay filter (order 3, number of coefficients 125).

For the compensation technique, at any wavelength λ, the absorbance (A) of a mixture of two species X and Y (which do not interact with each other) is governed by the law of absorbance additivities, A_m_ =A_X_ +A_y_. If the absorption of Y is subtracted from A_m_, the absorption characteristics of the mixture gradually approach that of X as C_Y_ increases. Finally, the absorption curve of mixture coincides with the absorption curve of X at the end-point, for which C_Y_ used as subtrahend is exactly the concentration of Y in the mixture. For a pure substance, the absorbance ratio at two wavelengths is constant over a certain range of concentration (i.e. independent of concentration and whether another absorbing component is present). Thus, the identification of Y is based on this ratio i.e. the absorbance ratio of the mixture is equal to that of pure Y meaning the concentration of Y in the sample solution is equal to that of pure Y.

For the absorbance ratio technique, the principle is based on the linear relationship between the absorbancy ratio value of a binary mixture and the relative concentration of such a mixture. The quantification analysis of AMO and CLO in a binary mixture is performed using the following equations:

$$C_{1}\;=\;\frac{Q\;-\;b_{1}}{a_{1}}\frac{A_{\mathrm{iso}}}{a_{\mathrm{iso}}}\;\times \;10^{3}$$

$$C_{2}\;=\;\frac{Q\;-\;b_{2}}{a_{2}}\frac{A_{\mathrm{iso}}}{a_{\mathrm{iso}}}\;\times \;10^{3}$$

where Q=A/A_iso_

C_1_ and C_2_: concentrations of AMO and CLA, respectively

A_iso_: absorbance at isosbestic point

a_iso_: absorptivity at isosbestic point ($=\frac{A_{\mathrm{iso}}}{C_{1}+C_{2}}$)

a_1_: slope of regression equation ($Q\;\mathrm{versus}\;\frac{C_{1}}{C_{1}+C_{2}}$)

a_2_: slope of regression equation ($Q\;\mathrm{versus}\;\frac{C_{1}}{C_{1}+C_{2}}$)

b_1_ and b_2_: intercept values of these regression equations

A: absorbance of mixture solution measured at the mixture’s maximum wavelength

10^3^: conversion factor of concentration unit from mg/mL to µg/mL.

For the UV derivative techniques, first-order derivative and ratio spectra first-order derivative spectra were evaluated for possible simultaneous determination of AMO and CLO.

High performance liquid chromatogram (HPLC) analysis was performed on an Agilent 1100 Series Diode-Array-Detector chromatograph (Agilent Technologies, USA) at ambient temperature. An Apollo C_18_ (150 × 4.6 nm, 5µm) was used. All solutions were filtered through a 0.45 µm membrane filter before injection into the chromatograph. All solvents were filtered through a 0.45 µm Millipore filter and degassed in an ultrasonic bath.

### Reagents and standard solutions

Amoxicillin trihydrate, AMO (98.4%), cloxacillin sodium, CLO (98.3%), and all excipients were kindly provided by Pharbaco Central Pharmaceutical Joint-Stock Company (Vietnam). De-ionized doubly distilled water was used throughout. All reagents were of analytical grade.

Stock solutions of AMO and CLO (1000 µg/mL) were prepared in water. Standard series of solutions were prepared in 25 mL calibrated flasks by using the same stock solutions and all dilutions freshly made.

### Sample solutions

FACLACIN 2, brand-name drug commercially available in the domestic market (produced by Pharbaco Central Pharmaceutical Joint-Stock Company, Vietnam, containing 250 mg AMO and 250 mg CLO per capsule) was studied.

The contents of 10 capsules were accurately weighed and finely powdered in a mortar. A mass corresponding to one-tenth of a capsule was transferred to a 100 mL calibrated flask containing about 30 mL water, well shaken, and ultrasonicated for 15 min. After the dissolution process, the solution was filtrated in a 100 mL calibrated flask through Whatman grade No. 42 filter paper. The residue was washed three times with 10 mL water and the volume completed to 100 mL with water. The resulting solution was further diluted to 1:5 in 25 mL calibrated flask with the same solvent for UV spectrophotometric measurement.

## RESULTS AND DISCUSSION

### Method development

### Non-derivative UV spectrophotometric techniques

**Figure 2:**
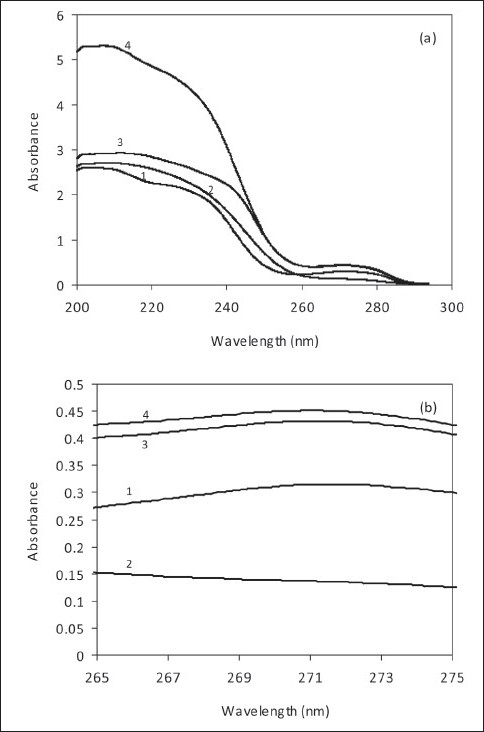
(a) Zero-order spectra of 100.0 µg/ml AMO (1), 100.0 µg/ ml CLO (2); spectrum of a mixture of 100.0 µg/ml AMO and 100.0 µg/ml CLO (3) and addition spectrum (4) of (1) and (2) in water; (b) corresponding zero-order spectra zoomed in

Figure 2(a) shows the zero-order UV absorption spectra in water indicating that the two spectra of AMO and CLO overlapped greatly in the wavelength region 200.0–300.0 nm. AMO exhibited a maximum at 271.8 nm, whilst CLO showed only a band of gradually reducing absorbance from 210.0 nm at 280.0 nm. Because the additivity of absorbances was not obeyed (as clearly shown in Figure 2b), the use of bivariate equation technique,[17] a.k.a Vierordt’s method,[18] selecting extrema of the two compounds was avoided due to error potentially acquired.

However, AMO and CLA could be still simultaneously determined using zero-order spectra such as compensation and absorbance ratio techniques. Unlike previously cited technique using derivative spectra,[19,20] the compensation technique in our study involves a comparison of several zero-order spectra (mixture – standard) using different concentrations of a standard solution as subtrahends. To determine the absorbance ratio at two selective wavelengths, a series of solutions containing different concentrations of pure drugs, above and below that presented in the binary mixture solution were analyzed [Table 1]. It is very important to mention that at the two wavelengths selected 250.2 and 252.5 nm, the mixture spectrum and addition spectrum coincided completely.

**Table 1:** Experimental parameters calculated for the simultaneous determination of AMO and CLO in binary mixture by compensation technique

Analyte	Concentration range (µg/ml)	Ratio	Mean (n=10)	RSD (%)
AMO	90.0-110.0	A_250.2_/A_252.5_	1.316	0.98
CLA	90.0-110.0	A_250.2_/A_252.5_	1.335	1.15

**Figure 3:**
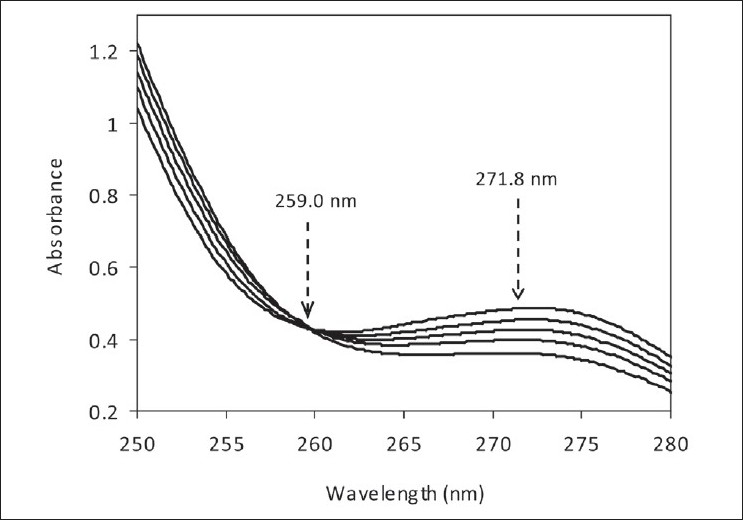
Zero-order spectra of 200.0 µg/ml binary mixtures containing AMO and CLO

On the other hand, binary mixtures containing AMO and CLA could be analyzed by the absorbance ratio technique[21] as described above. For this technique, the wavelengths for isosbestic point and mixture’s maximum absorbance were 259.0 and 271.8 nm, respectively [Figure 3].

Derivative UV spectrophotometric techniques

**Figure 4:**
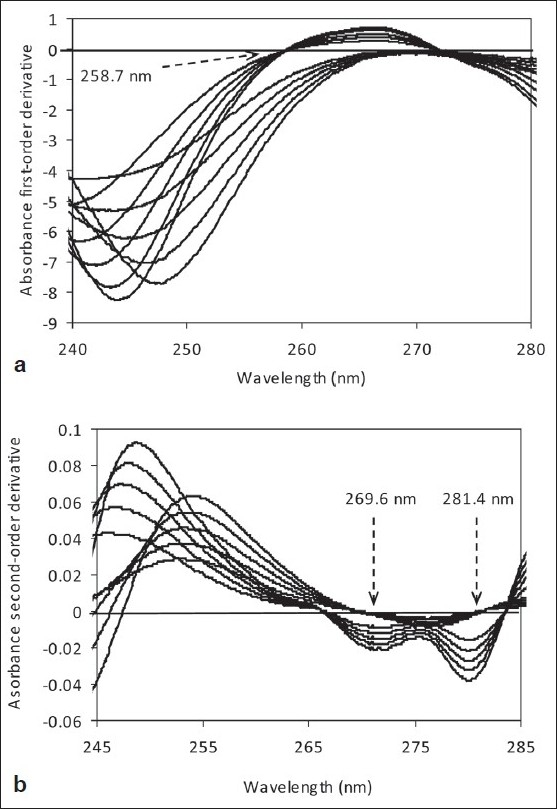
(a) First-order derivative spectra and (b) Second-order derivative spectra of AMO (60.0 – 140.0 µg/ml) and CLO (60.0 – 140.0 µg/ml) in water. The dashed arrows indicate working wavelengths

Figure 4(a) displays the first derivative spectra of these pure drugs revealing that there existed two zero-crossing points at 258.7 and 271.6 nm for AMO, whereas no zero-crossing point observed for CLO. Only 258.7 nm was chosen for the determination of CLO due to its derivative amplitudes at this wavelength proportional to the concentration range studied 60.07–140.0 µg/mL.

In contrast, Figure 4(b) shows two zero-crossing points at 269.6 and 281.4 nm for CLO. The second-order derivative values for AMO at these wavelengths, however, were not adequate for the correct determination of AMO from corresponding regression equations. This means AMO could not be determined using the UV first-order derivative technique.

**Figure 5:**
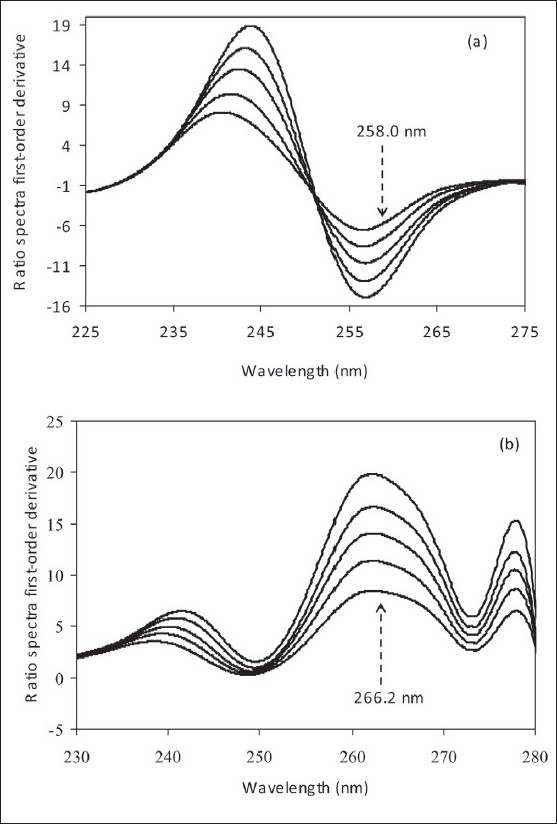
Ratio spectra first-order derivatives of (a) mixtures of 60.0 – 140.0 µg/ml AMO and 100.0 µg/ml CLO using a 60.0 µg/ml CLO as divisor and (b) mixtures of 60.0 – 140.0 µg/ml CLO and 100.0 µg/ml AMO using a 60.0 µg/ml AMO as divisor in water. The dashed arrows indicate working wavelengths

Figure 5 (a, b) present the first-order derivatives of zero-spectra used for the determination of AMO and CLO. To optimize this technique, the influence of divisor standard concentration was investigated with the concentration ranges for Lambert-Beer’s law compliance 60.0–140.0 µg/mL. A standard spectrum of 60.0 µg/mL was considered as suitable for the determination of both drugs. The determination of each component was based on the proportionality of its concentrations to relevant first-order derivative amplitudes at a suitable wavelength. 258.0 and 266.2 nm were chosen as working wavelengths for analyzing CLO and AMO, respectively.

**Figure 6:**
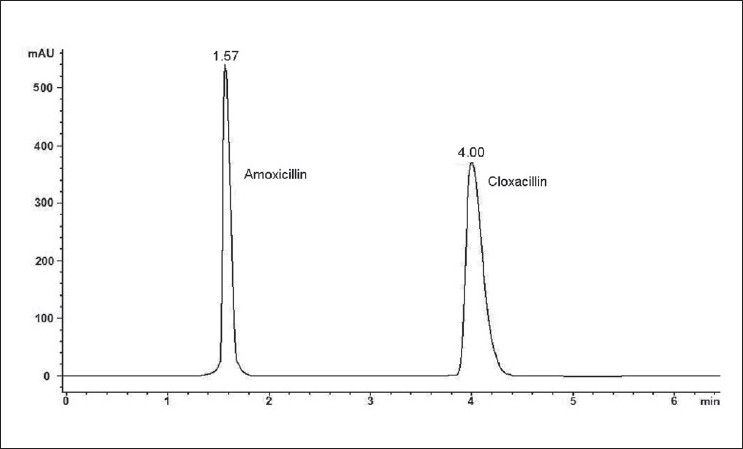
A typical chromatogram of a commercial sample (100.0 µg/ ml AMO and 100.0 µg/ml CLO)

In our study, the reversed-phase HPLC technique was developed to provide a specific procedure for the analysis of binary mixture containing AMO and CLO. The optimization of HPLC analysis was as follows. AMO and CLO were chromatographically analyzed by isocratic elution with a flow rate of 1.0 mL/min. The mobile phase composition was 0.01 M KH_2_PO_4_ – methanol (45:55, v/v). Injection volume was 20 µl and detection wavelength was 225.0 nm for both compounds. Under our chromatographic conditions, the retention times were found to be 1.57 and 4.00 min for AMO and CLO, respectively [Figure 6]. The chromatographic parameters such as resolution (Rs = 15.68), peak asymmetry (AF < 1.10), and plate number (960 / 15 cm) were satisfactory for both compounds obviously confirming the suitability of our HPLC technique.

### Method validation and application

The validity and suitability of the proposed UV spectrophotometric techniques were assessed by accuracy, precision, and linearity. For studying the accuracy, the proposed techniques were applied to simultaneously determine AMO and CLO in a synthetic mixture containing both the analytes and excipients which reproduced exactly the manufacturer’s formula. The amount of analyte recovered was expressed as average percent recovery with the upper and lower limits of standard deviation. The average percent recoveries obtained were 98.93 ± 0.95 and 101.04 ± 0.87% for AMO and CLO, respectively, indicating the techniques’ good accuracy and no marked interference by common excipients in the capsule studied. The within-run precision (repeatability) of these techniques was evaluated by analyzing six replicates of the synthetic mixture a day. The intermediate precision of these techniques was also evaluated with this mixture being analyzed during six consecutive days. The mean of relative standard deviation (%RSD = [S/X]×100, where S is standard deviation and X is mean of sample analyzed) were calculated. The low %RSD values (< 1.0%) of intra-day and inter-day variations indicate the proposed techniques’ good precision.

By analogy, the HPLC technique also showed good accuracy (99.86 ± 0.69 and 100.15 ± 0.71% recovered for AMO and CLO, respectively) and precision (%RSD < 0.5%) with the linearity ranges 60.0–140.0 mg/mL for both drugs.

The calibration graphs for HPLC and UV derivative spectrophotometric determination are summarized in Table 2.

**Table 2:** Calibration graphs of AMO and CLA by use of HPLC and UV derivative spectrophotometry

Technique	Analyte	Wavelength (nm)	Linearity range (µg/ml)	Linear regression equation	Squared correlation coefficient (R^2^)
HPLC	AMO	225.0	60.0 − 140.0	Y = 31.244C_AMO_ − 32.250	0.9995
	CLO	225.0	60.0 − 140.0	Y = 44.223C_CLO_ − 83.900	0.9996
First-order derivative	AMO	-	-	-	-
	CLO	258.7	60.0 − 140.0	Y = − 0.1500C_CLO_ − 0.0156	0.9997
Ratio spectra	AMO	258.0	60.0 − 140.0	Y = 0.1055C_AMO_ + 0.2635	0.9995
first-order derivative	CLO	266.2	60.0 − 140.0	Y = − 0.0954C_CLO_ + 0.1959	0.9998

The proposed techniques were successfully applied to the simultaneous determination of AMO and CLO in their combined capsules. The spectrophotometric results were statistically compared with those obtained by the HPLC technique [Table 3]. It is seen that at 95% confidence level, there was no significant difference between the accuracy (evaluated by the Student’s t-value) and precision (evaluated by the variance ratio F-value) between the spectrophotometric techniques and HPLC.

**Table 3:** Assay results for the determination of AMO and CLO in FACLACIN 2 capsule, and their comparison with HPLC

Analyte	Label claim (mg per tablet)	HPLC	Recovery ± SD % (n = 6) and their comparison with HPLC (p = 0.05, t = 2.228, F = 5.050)			
			First-order derivative	Ratio spectra first-order derivative	Absorbance ratio	Compensation
AMO	250	99.48±0.53	-	98.81±0.86	98.88±0.43	99.72±0.90
				t=1.633	t = 2.178	t = 0.566
				F=2.735	F = 1.462	F = 2.966
CLO	250	103.03±0.52	102.50±0.57	103.25±0.92	102.84±0.69	103.01±0.99
			t = 1.668	t = 0.507	t = 0.535	t = 0.044
			F = 1.157	F = 3.013	F = 1.695	F = 3.489

## CONCLUSION

In conclusion, the chromatographic separation of AMO and CLO in our study was characterized with good accuracy and precision. Except first-order derivative UV spectrophotometry, non-derivative techniques (i.e. absorbance ratio and compensation) and ratio spectra first-order derivative technique could be used for the simultaneous determination of AMO and CLO in their combined capsules. These UV spectrophotometric techniques using water as solvent were simple, reproducible, and accurate. Moreover, these techniques were statistically compared to chromatographic data suggesting possible interchangeability between UV spectrophotometric techniques and HPLC in the routine analysis.
